# Does Prior Breast Irradiation Increase Complications of Subsequent Reduction Surgery in Breast Cancer Patients? A systematic Review and Meta-Analysis

**DOI:** 10.1007/s00266-024-04038-6

**Published:** 2024-04-24

**Authors:** George Pappas, William Karantanis, Femi E. Ayeni, Senarath Edirimanne

**Affiliations:** 1 Department of Surgery, Nepean Hospital, Derby Street, Kingswood, NSW 2747 Australia; 2 Nepean Institute of Academic Surgery, Nepean Clinical School, The University of Sydney, 62 Derby Street, Kingswood, NSW 2747 Australia; 3 Faculty of Medicine, University of New South Wales, Sydney, NSW 2052 Australia

**Keywords:** Breast irradiation, Reduction mammoplasty, Mastopexy, Complications

## Abstract

**Background:**

Reduction mammoplasty and mastopexy are potentially complicated by prior breast irradiation as part of breast conserving therapy. Associated tissue changes with therapeutic irradiation have led to surgeons deciding the risks may outweigh potential benefit for those patients.

A systematic review of the existing literature was performed to explore surgical outcomes of patients undergoing delayed bilateral reduction mammoplasty or mastopexy following unilateral breast irradiation as part of breast conserving therapy.

**Methods:**

Medline, PubMed and EMBASE were searched from 1990 to 2023 according to PRISMA guidelines. Studies were combined by the generic inverse variance method on the natural logarithms of rate ratios (RR) using a random effect model in Review manager 5.4.1.

**Results:**

Fifteen studies reported outcomes in 188 patients who underwent breast reduction (BR) following unilateral breast conserving surgery and radiotherapy. The median age at BR was 51.5 years (range 39–60), and median time since radiotherapy was 48 months (range 11.7–86). We compared outcomes for irradiated breast (IB) versus non-irradiated breast (NIB). Pooled results showed higher rate of major complications in the IB (RR 2.52, 95%CI 0.96–6.63, *p*=0.06), but not statistically significant. However, rate of minor complications was significantly higher in the IB (RR 3.97 95%CI 1.86-8.50, *p*<0.0004). Incidence of fat necrosis as a discrete complication was 2× higher in IB (RR 2.14 95%CI 0.85–5.35, *p*-value 0.10) compared to the NIB, but not significant.

**Conclusion:**

We found breast reduction to be safe with acceptable risk of major complications. However, the overall complication rate remains higher in IB compared to NIB.

**Level of Evidence III:**

This journal requires that authors assign a level of evidence to each article. For a full description of these Evidence-Based Medicine ratings, please refer to the Table of contents or the online Instructions to Authors www.springer.com/00266.

**Supplementary Information:**

The online version contains supplementary material available at 10.1007/s00266-024-04038-6.

## Introduction

Breast cancer is the leading cause of cancer-associated deaths in women worldwide. Improved diagnostic methods and screening programmes increasingly enable breast cancer diagnosis at an early stage, facilitating early intervention [1]. Breast conservation therapy (BCT), a combination of Wide local excision and radiation therapy (RT), is the cornerstone for early-stage breast cancer treatment [2, 3]. However, unilateral resection of breast tissue and irradiation leads to significant breast asymmetry and other aesthetic complications, such as scars [4–6]. Hence, BCT poses significant psychological stress to this patient population [7, 8].

This aesthetic deformity is treated with a reduction mammoplasty or mastopexy, gold standard procedures for surgical management of breast asymmetry. These procedures aim to alleviate psychological stress and physical trauma associated with breast asymmetry, thereby improving the quality of life of these patients [9, 10].

However, breast RT results in dose-dependent early and late pathophysiological changes to the skin, its microenvironment and breast parenchyma [11]. This includes endothelial dysfunction and vessel compromise in both tissue microvasculature and macrovasculature, stromal fibrosis and activation of pro-inflammatory cascades [12]. Many of these histologic changes are beneficial in oncological treatment, but may result in post-radiation complications. These effects are often long-lasting and may further complicate reoperation on irradiated breast tissue [13, 14].

To date, only few studies had reported outcomes of bilateral mammoplasty following unilateral BCT. Handel et al. in 1992 were among the first to report post-radiation reduction mammoplasty. Complications in the irradiated breast (IB) included prolonged erythema, significant necrosis of the nipple, loss of partial thickness of the areola, nipple–areola pigmentation loss and delayed healing after free nipple graft reduction mammoplasty [15]. The non-irradiated breast (NIB) experienced no complication. To date, there remains a paucity of data available about the outcome of bilateral reduction surgery addressing immediate or delayed post-surgical complications following breast RT in both the IB and NIB. Thereby, surgeons are careful while performing post-radiation reduction mammoplasty.

This systematic review considers the published literature regarding bilateral reduction mammoplasty following unilateral breast RT. The goal is to explore whether irradiation of the breast increases risk of complication after reduction mammoplasty and the association of complication rates with the timing of reduction mammoplasty post-radiation.

## Materials and Methods

### Search Strategy

We conducted this systematic review following recommended guidelines of Preferred Reporting Items for Systematic Review and Meta-Analysis (PRISMA) [16]. The goal of this search was specifically literature detailing cases of bilateral reduction mammoplasty (or mastopexy) following unilateral breast RT, to compare the IB and NIB. We performed a literature search on PubMed, Medline and EMBASE databases in January 2023. Keywords used were “breast conservation therapy,” “reduction mammoplasty,” “breast reduction,” “mastopexy,” “irradiated breast” and “asymmetry correction.”

### Inclusion Criteria

The eligibility criteria for the studies for inclusion for systematic review were outlined in Table 1.

**Table 1:** Inclusion criteria

Subjects	Patients who had a bilateral reduction mammoplasty or mastopexy following unliteral breast irradiation for BCT
Outcomes	Complication rate Mass of tissues reduction Cosmetic outcomes Complication rate Reintervention rates for complications
Publication year	1990–2023
Study design	Case reports, case series and retrospective studies were all included in this review. Studies with less than five patients were included in this review.
Study language	English

Animal studies, review articles and studies which were published in languages other than English were excluded from the review.

### Data Extraction

Two reviewers (GP and WK) independently extracted relevant data from included articles. No automated extraction software was utilised. Outcomes observed were divided into demographic, aesthetic and complication outcomes for both the irradiated breast (IB) and non-irradiated breast (NIB). Demographic outcomes included: number of reduction mammoplasty, number of mastopexy, surgical technique, specimen mass, follow-up, age, radiation dose, RT surgery interval, BMI, smoking and comorbidities. Aesthetic outcomes included: incidence of scar issues, clinician/patient-reported asymmetry post-operatively and reintervention for aesthetic complication. Complication outcomes were: fat necrosis, infection, nipple–areolar complex loss, skin necrosis, seroma, haematoma, wound dehiscence and reintervention for complication. Complication data were collected as both “total complications” and separately as major and minor complications. Major complications were defined in accordance with the Clavien–Dindo classification for surgical complication [17]. This classification defines Grade 3 complications as those requiring surgical, endoscopic or radiologic interventions. Hence, in this paper we ascribed the term “major” to imply complications necessitating further intervention—radiologic or surgical—and “minor” complications to mean those that did not require further intervention (Clavien–Dindo Grade 1/2).

### Statistical Analysis and Data Synthesis

For data synthesis and presentation, results were reported as a mean for numerical data or range and rates for categorical parameters. There were no ordinal data points collected. Where applicable, we carried out unpaired *t*-test to compare whether there was a significant difference in the mean between two groups using GraphPad prism. Complication counts as outcomes were treated as count data. There were multiple problematic zero count complications, so there was zero count correction by the addition of fixed 0.5 to cells. The studies were combined by the generic inverse variance method on the natural logarithms of rate ratios (RR) using a random effect model in Review manager 5.4.1. Rate ratios (RR) were reported with 95%confidence intervals, and significance level was set at *p* < 0.05.

### Quality Assessment

Quality assessment of included papers was performed using the Methodological Index for Non-Randomized Studies (MINORS) tool. This is a validated tool for non-randomised surgical studies, both comparative and non-comparative, with numerical scores 0 (not reported), 1 (inadequately reported) or 2 (adequately reported) for eight different study parameters (Table 7). This equates to a maximum score of 16 [18].

## Results

### Study Selection

**Fig. 1:**
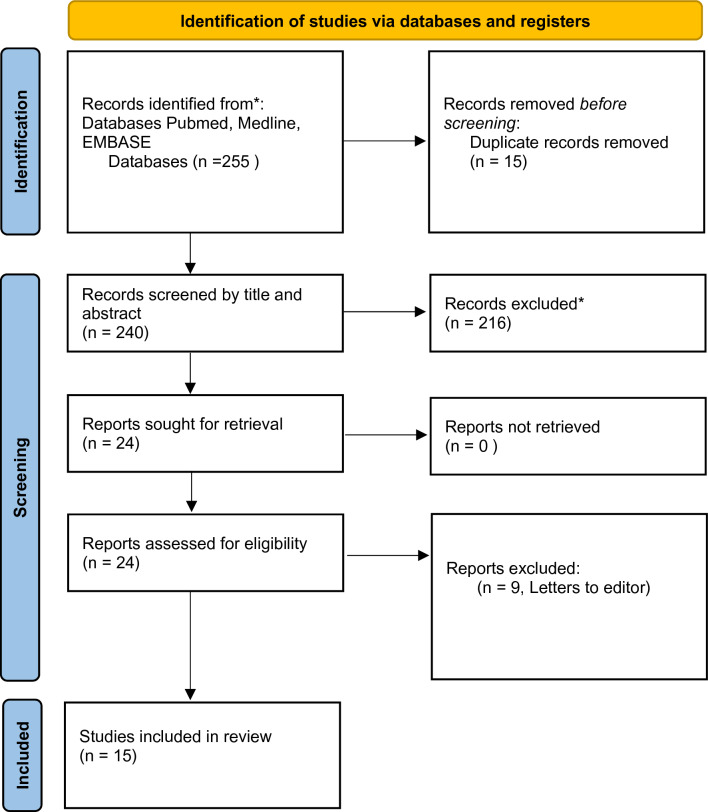
Flow chart of papers included in study.

The search yielded a total of 255 articles, with 15 duplicates found. A total of 240 articles were screened via title and abstract, and 216 were excluded as they did not meet the inclusion criteria or were unrelated to the topic. Twenty-four papers were retrieved and nine removed as they were letters to the editor (Fig. 1). Hence, a total of fifteen studies met the eligibility criteria and were included in the systematic review [4, 15, 19, 22, 23, 29, 30, 32–34, 36, 40, 43, 44], but two studies [30, 34] did not have data reported for some of the outcomes, and were not included in the meta-analysis. All studies selected were published between 1991 and 2023 (Table 2). The observational studies were published across 6 different nations with USA representing 66.7% (*n*=10) of papers published.

**Table 2:** Study characteristics and patient demographics. Data represented as mean (range) +/− standard deviation.

Study	Period	Location	No. of Patients	No. of Reduction mammoplasty	No. of Mastopexy (<100g)	Surgical Technique	Mean specimen mass		Interval between radiotherapy and surgery (months)	Mean radiation dose (Gy)	Mean post-op follow-up (months)	Mean age	Smoking history
							IB	NIB					
Handel et al, [15]	1989–1992	Van Nuys, California, USA	1	1	0	Bilateral reduction mammoplasty with full thickness NAC graft (*n*=1)	965	940	18	66 (66–66)	12 (12–12)	53.0 (53–53)	NR
Spear et al, [32]	1995–1998	Washington D.C, USA	3	3	0	Modified Bilateral McKissock (*n*=1) Standard McKissock (NIB) with Superior pedicle reduction (IB) (*n*=1) Bilateral Inferior Pedicle (*n*=1)	596.7	826.7	11.7 (3–24)	60.5 (60–61)	NR	46.3 (45–48)	NR
Kronowitz et al, [23]	1990–2006	Texas, USA	8	8	0	Inferior pedicle (*n*=6) Superior pedicle (*n*=1) Central pedicle (*n*=1)	NR	NR	37	NR	NR	NR	NR
Tuncer et al, [22]	2005–2006	Ankara, Turkey	1	1	0	Inferior pedicle technique (*n*=1)	600	750	30 (30–30)	NR	7 (7–7)	39 (39–39)	0
Christiansen et al, [4]	2001–2008	Columbia, Missouri, USA	5	5	0	IB: Omega reduction technique (*n*=5) NIB: Wise pattern, inferior pedicle, central mould technique (*n*= 5)	291	532.8	56 (36–84)	NR	NR	50 (41–56)	NR
Chin et al, [33]	1997–2009	Boston, MA, USA	12	7	0	IB: Wise pattern inferior pedicle (*n*=5) Breast amputation with free nipple graft (*n*=2) NIB: “Corresponding corrective procedure” (*n*=7)	892	NR	78	59.7	10	49	0
				0	6	IB: Vertical/T-shaped mastopexy (*n*=6), NIB: “corresponding corrective procedure” (*n*=6)	230	NR	52	61.6	13	48.0	0
Parret et al, [19]	2004–2010	Boson, MA, USA	12	12	0	Robertson reduction (*n*=7) Short-scar periareolar inferior pedicle reduction (*n*=3) Wise pattern reduction (*n*=2)	306	599	86 (14–132)	55	10	57 (47–64)	0
Patel et al, [34]	2003–2010	Washington D.C, USA	5	5	0	Inferior pedicle (*n*=1) Superomedial pedicle (*n*=1) Central pedicle (*n*=2) Medial pedicle (*n*=6)	397.4	NR	NR	NR	50.4 +/− 22.5	56 +/− 13.7	1
Munhoz et al, [29]	1999–2011	Sao Paolo, Brazil	38	38	0	Superior medial (*n*=17) Superior (*n*=13) Inferior (*n*=6) Superior lateral (*n*=2)	NR	NR	22 (14–48)	55–60	NR	51.5 (39–66)	8
Dal Cin et al, [43]	1980–2012	Hamilton, Ontario, Canada	9	9	0	Inferior pedicle (*n*=8) Superior pedicle (*n*=1)	490.4	664.1	65 +/−56	50	32.9 (4–84)	56.2 +/− 9.2	NR
Spear et al, [45]	1995–2014	Washington D.C, USA	18	12	6	Mammoplasty techniques- Inferior, medial, central mound, superomedial, superolateral and McKissock pedicles Mastopexy techniques - Circumvertical, inferior wedge excision and free nipple graft	623	NR	30	NR	26.3 (1.8–119.3)	49.5 (35–69)	1 (former)
Egro et al, [30]	2005–2015	Atlanta, GA, USA	25	25	0	NR	425.4	648.8	60.7 +/− 43.5	NR	44.8 +/− 22.1	56.1 +/− 8.5	2
Weichman et al, [36]	2008–2015	New York, USA	13	13	0	Central mound technique (*n*=13)	254.1	386.9	41.3 (9–132)	60	NR	50.2	6 (former)
Barnea et al, [44]	2009–2018	Tel-Aviv, Israel	25	11	14	Approach varied based on lumpectomy site.	175	451	48.0 (6–180)	NR	8.5 (6–24)	60.8 (42–74)	5
Prasidha et al, [40]	2009–2023	Sydney, NSW, Australia	13	10	0	IB: Inferior pedicle (*n*=7) Superomedial pedicle (*n*=3) NIB: Inferior pedicle (*n*=10)	541	743.6	50.4 (24–108)	NR	9.1 (1–25)	59.5 (41–78)	0
				0	3	IB: Skin de-epithelialisation only (*n*=3) NIB: LeJour pattern mastopexy (*n*=2) Inferior pedicle reduction mammoplasty (*n*=1)	NR	NR	49.2 (12–120)	NR	16.3 (11–26)	57.6 (46–65)	2

### Study and Patient Characteristics

A total of 188 patients were analysed in the systematic review. 84.7% (*n*=160) patients underwent reduction mammoplasty in the irradiated breast compared to 15.3% (*n*=29) patients who had mastopexy in the IB. One patient required a revision mammoplasty, accounting for 189 surgical procedures in 188 patients. A range of surgical procedures were reflected in the published literature (Table 2). Mean age of the participants included was 52yrs (± 5.6).

Mean duration between surgical resection and termination of radiation therapy was 45.7 months (±21.5), and mean post-surgical follow-up was 20.0 months (±15.1). Mean resection weight in the non-irradiated breast (NIB), 654.3g (±169.7), was greater than that of the irradiated breast (IB), 484.8g (± 238.5), but was not statistically significant, *p*=0.068 (Table 3).

**Table 3:** Aesthetic outcomes and reintervention following reduction mammoplasty or mastopexy.

Study	No. of patients	Scarring issues		Clinician or patient-reported breast asymmetry post-reduction	Number of revision procedures for asymmetry or scarring issue post-reduction	Reoperated breast and procedure performed
		IB	NIB			
Handel et al, [15]	1	0	0	1	0	0
Spear et al, [32]	3	Inframammary dog ear (*n*=1)	0	0	1	Excision of inframammary dog ear in rooms (*n*=1)
Kronowitz et al, [23]	8	NR	NR	NR	NR	NR
Tuncer et al, [22]	1	0	0	1	0	0
Christiansen et al, [4]	5	0	0	1	1	IB, revision reduction (*n*=1)
Chin et al, [33]	12	Minor scarring issues (*n*=1)	0	1	1	Revision mastopexy (*n*=1)
Parret et al, [19]	12	NR	NR	3	2	NR
Patel et al, [34]	5	NR	NR	NR	NR	NR
Munhoz et al, [29]	38	NR	0	NR	3	NR
Cin et al, [43]	9	3	0	7	3	NIB repeat reduction mammoplasty (*n*=3)
Spear et al, [45]	18	NR	NR	NR	NR	NR
Egro et al, [30]	25	2	NR	6	NR	NR
Weichman et al, [36]	13	1	3	NR	NR	NR
Barnea et al, [44]	25	NR	NR	NR	NR	NR
Prasidha et al, [40]	13	NR	NR	NR	NR	NR
		NR	NR	NR	NR	NR

### Surgical Complications in the Irradiated (IB) and Non-Irradiated Breast (NIB)

A total of 103 complications were reported in 188 patients and 189 procedures as one patient required bilateral revision mastopexy. 92.2% (*n*=95) of these complications occurred in the irradiated breast and 7.8% (*n*=8) in the non-irradiated breast (Table 4). To characterise the rate of complication for individual patients, the number of patients who experienced any complication was recorded (Table 4). These data were not included in 3 studies. However, in studies that did record this information, accounting for 162 patients, only 32 patients experienced any form of complication (major or minor) accounting for an overall complication rate of 19.8%. There were 15 revision procedures documented for surgical complications. The procedures ranged from seroma drainage to extensive flap reconstruction. Few studies (n=6) reported histologic outcomes following delayed reduction mammoplasty; however, these are documented in Table 4. DCIS and tumour recurrence were the most common abnormal histologic findings, reinforcing the need to ensure all intraoperative specimens are analysed and the results reviewed.

**Table 4:** Summary of total complications (major and minor), adverse outcomes and revision procedures for complication.

Study	No. of patients	Total complications (Sum of IB /NIB in row)		Number of breasts that experienced any complication (excluding asymmetry)		Number of patients that experienced a surgical complication	Post-operative antibiotics given for infection (All for IB)	Number of revision procedures or interventions for complications		Revision procedure performed for complications	Abnormal histologic findings from IB
		IB	NIB	IB	NIB			IB	NIB		
Handel et al, [15]	1	4	0	1	0	1	1	0	0	Nil	Atrophic non-proliferative breast tissue (*n*=1)
Spear et al, [32]	3	1	0	1	0	1	0	0	0	Nil	Fibrous mastopathy (*n*=1), Fibrocystic change and apocrine metaplasia (*n*=1), Stromal fibrosis (*n*=1)
Kronowitz et al, [23]	8	7	0	4	0	4	NR	NR	NR	NR	NR
Tuncer et al, [22]	1	0	0	0	0	0	0	0	0	Nil	Nil
Christiansen et al, [4]	5	1	1	1	1	1	0	0	0	Nil	NR
Chin et al, [33]	12	2	0	2	0	2	0	NR	NR	NR	Recurrent ductal carcinoma in situ (*n*=1)
Parret et al, [19]	12	10	0	5	0	5	2	6	0	6. Irrigation, debridement and reoperation (n=2), Seroma drainage (n=6)	NR
Patel et al, [34]	5	3	0	3	0	3	NR	NR	NR	NR	Nil
Munhoz et al, [29]	38	18	2	12	2	12	2	5	0	Flap advancement (*n*=5)	NR
Dal Cin et al, [43]	9	11	0	NR	NR	NR	NR	0	0	Nil	Ductal carcinoma in situ (*n*=2)
Spear et al, [45]	18	12	0	5	0	0	NR	1	0	Latissimus dorsi musculocutaneous flap reconstruction (*n*=1)	NR
Egro et al, [30]	25	11	NR	NR	NR	NR	NR	NR	NR	NR	Recurrence (*n*=4)
Weichman et al, [36]	13	4	3	NR	NR	NR	NR	1	0	Biopsy of fat necrosis (*n*=1)	Nil
Barnea et al, [44]	25	10	0	7	0	0	NR	2	0	Debridement and primary closure (*n*=1), Autologous deep inferior epigastric perforator flap (*n*=1)	Recurrent breast cancer (*n*=1)
Prasidha et al, [40]	13	1	2	1	2	3	0	0	0	Nil	Nil
		0	0	0	0	0	0	0	0	Nil	Nil

**Fig. 2:**
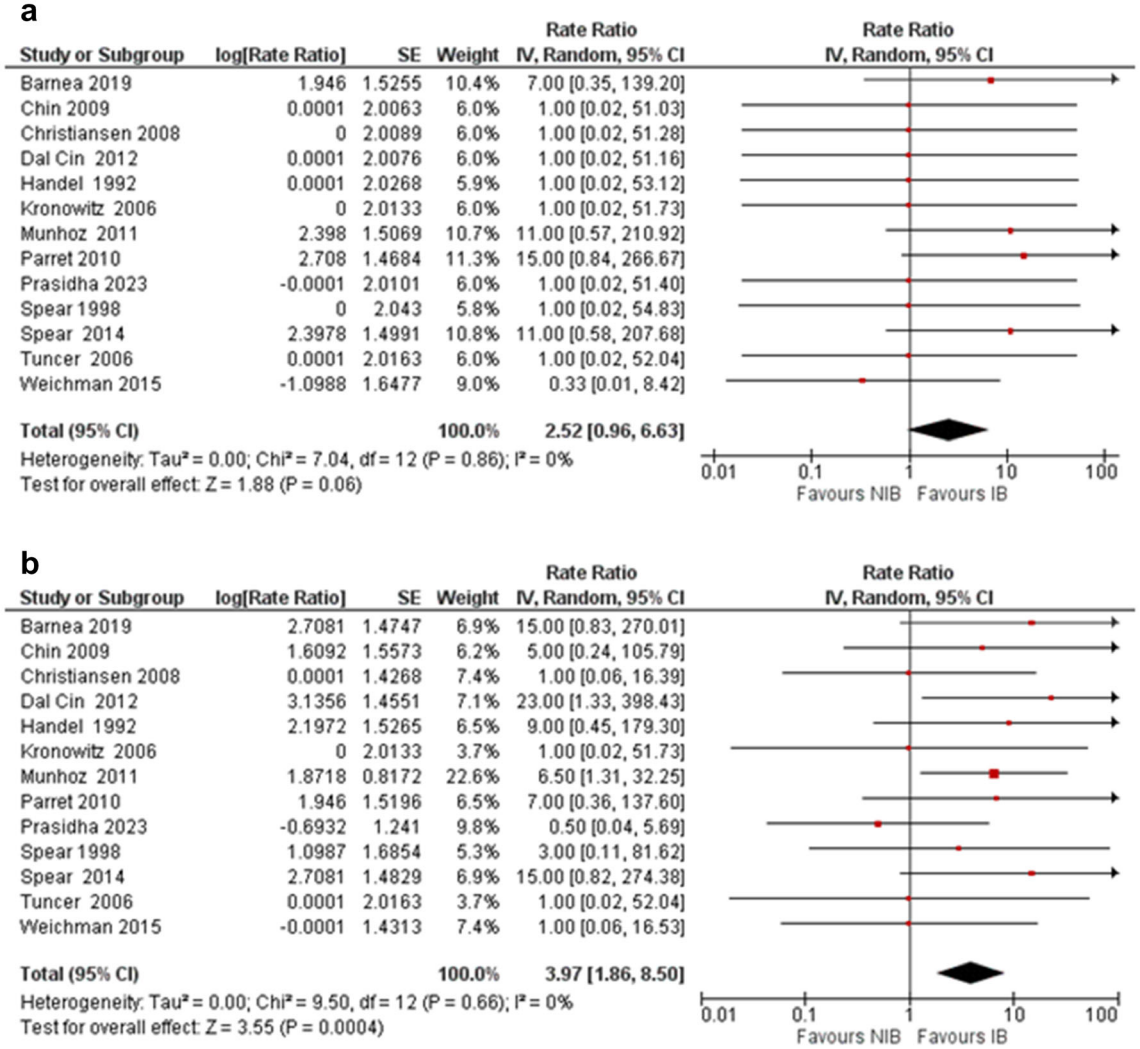
**a** Forest plot depicting major complications in the irradiated and non-irradiated breast. **b** Forest plot depicting minor complications in the irradiated and non-irradiated breast.

There were 21 total major complications noted in this systematic review (Table 5). These complications accounted for 20.4% of the overall complications. Only one of these complications, a haematoma requiring evacuation, occurred in a non-irradiated breast. The most common major complications were skin necrosis requiring reoperation (*n*=5) and seroma requiring intervention (*n*=5). Pooled results showed higher complication rate of major complications in the irradiated breast (RR 2.52, 95%CI 0.96-6.63), but this was not statistically significant, *p*=0.06 (Fig. 2a).

**Table 5:** Summary of major complications

Study	Infection (requiring debridement)		Fat necrosis (requiring debridement)		NAC loss (requiring reoperation)		Skin necrosis (requiring intervention)		Seroma (requiring reoperation)		Haematoma		Wound dehiscence (requiring reoperation)		Total major complications	
	IB	NIB	IB	NIB	IB	NIB	IB	NIB	IB	NIB	IB	NIB	IB	NIB	IB	NIB
Handel et al, [15]	0	0	0	0	0	0	0	0	0	0	0	0	0	0	0	0
Spear et al, [32]	0	0	0	0	0	0	0	0	0	0	0	0	0	0	0	0
Kronowitz et al, [23]	0	0	0	0	0	0	0	0	0	0	0	0	0	0	0	0
Tuncer et al, [22]	0	0	0	0	0	0	0	0	0	0	0	0	0	0	0	0
Christiansen et al, [4]	NR	NR	0	0	0	0	0	0	NR	NR	NR	NR	0	0	0	0
Chin et al, [33]	0	0	0	0	0	0	0	0	NR	NR	NR	NR	NR	NR	0	0
Parret et al, [19]	0	0	1	0	0	0	0	0	5	0	NR	NR	1	0	7	0
Patel et al, [34]	NR	NR	NR	NR	NR	NR	NR	NR	NR	NR	NR	NR	NR	NR	NR	NR
Munhoz et al, [29]	0	0	0	0	0	0	4	0	NR	NR	0	0	1	0	5	0
Dal Cin et al, [43]	0	0	0	0	0	0	NR	NR	0	0	0	0	NR	NR	0	0
Spear et al, [45]	1	0	1	0	1	0	1	0	0	0	0	0	1	0	5	0
Egro et al, [30]	0	NR	0	NR	0	NR	0	NR	0	NR	0	NR	0	NR	0	NR
Weichman et al, [36]	0	0	0	0	0	0	NR	NR	NR	NR	0	1	NR	NR	0	1
Barnea et al, [44]	1	0	2	0	NR	NR	NR	NR	NR	NR	NR	NR	0	0	3	0
Prasidha et al, [40]	0	0	0	0	0	0	NR	NR	NR	NR	NR	NR	NR	NR	0	0
	0	0	0	0	0	0	NR	NR	NR	NR	NR	NR	NR	NR	0	0

We noted the incidence of fat necrosis was two times higher in the irradiated breast (CI 0.85–5.35, *p*-value 0.10) versus the non-irradiated breast (Supp Fig. 1)

Similarly, there were 51 total minor complications observed in the literature analysed (Table 6), of which 45 (89.5%) of these complications occurred in the IB. Complications in the NIB included delayed wound healing (*n*=3), skin necrosis (*n*=1), oedema (*n*=1) and wound dehiscence (*n*=1). The most common minor complications in the IB were infection (*n*=14) and delayed wound healing (*n*=10). Meta-analysis (Fig. 2b) showed that complication rate of minor complications was significantly higher in the IB (RR 3.97 95%CI 1.86–8.50, *p*<0.001). Given the classification of major and minor complications in this paper, we found 25 complications that could not be categorised as “major” or “minor” (Table 6).

**Table 6:** Summary of minor complications

Study	Infection (not requiring debridement)		Fat necrosis (not requiring debridement)		NAC Loss (not requiring reoperation)		Skin necrosis (not requiring reoperation)		Seroma		Delayed wound healing		Wound dehiscence (treated conservatively)		Oedema		Other complications		Total minor complications		Cases where distinction where major v minor complication unclear
	IB	NIB	IB	NIB	IB	NIB	IB	NIB	IB	NIB	IB	NIB	IB	NIB	IB	NIB	IB	NIB	IB	NIB	
Handel et al, [15]	1	0	0	0	1	0	0	0	0	0	1	0	0	0	0	0	Depigmentation of grafted NAC (*n*=1)	0	4	0	0
Spear et al, [32]	0	0	0	0	0	0	0	0	0	0	0	0	0	0	1	0	0	0	1	0	0
Kronowitz et al, [23]	0	0	0	0	0	0	0	0	0	0	NR	NR	0	0	0	0	0	0	0	0	7. Infection (*n*=1), fat necrosis (*n*=1), nipple–areola loss (*n*=1), wound dehiscence (*n*=1), seroma (*n*=3)
Tuncer et al, [22]	0	0	0	0	0	0	0	0	0	0	0	0	0	0	0	0	0	0	0	0	0
Christiansen et al, [4]	NR	NR	0	0	0	0	0	0	NR	NR	0	0	0	0	1	1	0	0	1	1	0
Chin et al, [33]	0	0	0	0	0	0	0	0	NR	NR	1	0	NR	NR	1	0	0	0	2	0	0
Parret et al, [19]	2	0	1	0	0	0	0	0	0	0	NR	NR	0	0	NR	NR	0	0	3	0	0
Patel et al, [34]	NR	NR	NR	NR	NR	NR	NR	NR	NR	NR	NR	NR	NR	NR	NR	NR	NR	NR	NR	NR	3. Unspecified complications
Munhoz et al, [29]	2	0	4	0	1	0	3	1	NR	NR	NR	NR	3	1	NR	NR	0	0	13	2	0
Dal Cin et al, [43]	6	0	0	0	1	0	NR	NR	0	0	2	0	NR	NR	NR	NR	Secondary infection (*n*=2)	0	11	0	0
Spear et al, [45]	1	0	0	0	0	0	1	0	0	0	4	0	0	0	0	0	Prolonged induration (*n*=1)	0	7	0	0
Egro et al, [30]	0	NR	0	NR	0	NR	0	NR	0	NR	0	NR	0	NR	NR	NR	0	NR	0	NR	11. Infection (*n*=4), fat necrosis (*n*=2), nipple–areola loss (*n*=1), seroma (*n*=1), wound dehiscence (*n*=1), delayed healing (*n*=2)
Weichman et al, [36]	0	0	0	0	0	0	NR	NR	NR	NR	1	1	NR	NR	NR	NR	0	0	1	1	4. Infection (*n*=2), fat necrosis (*n*=2)
Barnea et al, [44]	2	0	2	0	NR	NR	NR	NR	NR	NR	NR	NR	2	0	NR	NR	Nipple congestion (*n*=1)	0	7	0	0
Prasidha et al, [40]	0	0	0	0	0	0	NR	NR	NR	NR	1	2	NR	NR	NR	NR	0	0	1	2	0
	0	0	0	0	0	0	NR	NR	NR	NR	0	0	NR	NR	NR	NR	0	0	0	0	0

### Aesthetic Outcomes

Aesthetic outcomes in this systematic review were defined as issues relating to scars or asymmetry. Interestingly only 8 (53.3%) studies considered symmetry in their results (Table 3). Of these studies, 20 patients of 68 (29.4%) had asymmetry described by either patient or clinician following reduction mammoplasty or mastopexy. Ultimately, 11 patients underwent revision procedures for aesthetic outcomes.

### Quality Assessment

The mean MINORS score for the included studies was 8.5 (range 3–11). Given the included studies were all retrospective in nature, all studies score 0 for prospective sample size calculation. Given the assessment of symmetry and complication can be biased and vary between clinicians, most included studies also score 0 in this domain.

## Discussion

In this systematic review, we outline the post-operative complications and aesthetic outcomes after delayed bilateral reduction mammoplasty and mastopexy, following unilateral radiation therapy for BCT IB in breast cancer patients. The main outcome of this study reaffirms the higher rate of post-operative complications in the previously IB when compared to NIB. These complications vary in severity with some complications resolving with conservative, non-invasive interventions while others tend to require a reoperation/invasive drainage procedures [19].

Changes induced in the breast after radiation are indicative of complications that would occur in case of any future surgery. Post-radiation changes are biphasic. In the first phase, after a few days or within a week of radiation, cell death of rapidly proliferating cells occurs. Furthermore, radiation-induced inflammation leads to necrosis, tissue atrophy and chronic ulceration of the breast tissue [13, 20, 21]. Another noteworthy post-radiation change is reduced skin perfusion which may last up to a year [22–25]. During the later stage, which is within a few months or a year, adipose tissue is replaced with collagen, accompanied by a reduction of elastic components, collagen fibre hyalinisation, epidermis thinning and the basal membrane of blood vessels get thickened. These permanent fibrotic changes are responsible for post-surgical complications [22]. Some studies detail tissue ischaemia in irradiated tissue, although this remains challenged in published data [26, 27].

Rudolph (2015) argues post-radiation complications are not due to tissue ischaemia but rather by the poor responding ability of tissues to surgical injury and tension on wound closure due to permanent stem cell and intrinsic fibroblast deficiency which are caused by irradiation [28].

Although breast surgeons are well aware of complications associated with operating on irradiated tissues, the increasing number of patients presenting for breast reduction surgery and the high rate of post-surgical complications, compel surgeons to take precautions in preoperative planning. In this regard, some authors have suggested the timing of reduction surgery as the determining factor for post-surgical complications [29, 30].

### Aesthetic Outcomes

An acceptable aesthetic outcome is one of the central tenants of any reduction mammoplasty or mastopexy [31]. Of the included studies ten reported some form of unsatisfactory aesthetic outcome, some due to scarring or post-reduction asymmetry [15, 22, 32, 33] . Of the included published papers, five demonstrated the effect of timing on the aesthetic outcomes of reduction surgery. Delayed reconstruction was associated with a higher risk of poorer aesthetic outcomes [34]. Indeed Patel et al. reported worse aesthetic outcomes in the delayed reduction group as compared to immediate and staged-immediate reduction; however, they were not statistically significant. Interestingly, similar score values were found for patient satisfaction and quality of life for the three groups using the BREAST-Q questionnaire [34]. This may be due to certain patient factors, for example, a patient may be satisfied that they have avoided a mastectomy and accept some degree of ongoing asymmetry. It may also relate to the immediate physical benefit that reduction mammoplasty achieves, which maybe an important factor that determines patient satisfaction as compared to asymmetry.

Apart from better aesthetic outcomes, immediate oncoplastic reduction at the time of lumpectomy confers the advantage of single-stage operative intervention as opposed to multiple admissions and anaesthesia. Furthermore, the tumour is included in the tissue being removed for reduction potentially providing greater exposure and access to the oncological mass and extensive resection, although there is the risk of positive margins being included in the reduction specimens and possible risk of mastectomy. Complete removal of the tumour by reduction has been reported [19, 31, 35].

### Complications

The surgical techniques employed for reduction mammoplasty or mastopexy have been reported to affect the occurrence of post-surgical complications. In a case series by Katie et al., a similar rate of complications was found in IB and NIB after reduction. The authors concluded that this was attributed to the surgical technique employed, i.e. central mound with minimal elevation of skin/devascularisation. Thus, the authors suggested that the central mound technique is reliable for the reduction mammoplasty or mastopexy for asymmetry/macromastia after radiation [36]. Interestingly, in this review, two studies did not find any significant difference in post-radiation complication rates between IB and NIB [32, 33].

The result of this meta-analysis indicates that the risk of minor complications post-reduction mammoplasty in the irradiated breast were more common. In a cohort of 188 patients, 51 complications were recorded in the IB and 6 in the NIB. Major complications included nipple–areola complex loss, fat necrosis requiring debridement, skin necrosis, haematoma and infections requiring debridement and drainage.

Due to the inherent risk of post-operative complications in irradiated tissue, attempts have been made to reduce these complications through prophylactic treatment. Perioperative antibiotics have been used to combat infections after breast reduction surgery. Nevertheless, it is not recommended to administer antibiotics as prolonged prophylactic treatment due to resistance issues and lack of evidence [37]. Decreased vascularisation in irradiated breast tissue has a role in complication occurrence, and a vasoactive agent, buflomedil, has been administered to patients intravenously and then per oral for 14 days to prevent complication [38]. However, further studies are required in this regard as this was the only study that reported the use of buflomedil in a patient undergoing breast reduction surgery [32].

In another study, pre- and post-operative adjuvant hyperbaric oxygen therapy was given to the patients undergoing reduction mammoplasty or mastopexy after BCT [39]. Apart from delayed wound healing in two irradiated and non-irradiated breasts, no other complications were reported. But the clinical use of this technique is limited owing to chamber accessibility. Hyperbaric oxygen therapy might help to reduce the risk of complications by increasing angiogenesis and vascularisation [39]. This topic has been previously considered in a systematic review and meta-analysis by Lorentzen et al., 2021 [37, 40]. The study concluded that reduction mammoplasty or mastopexy in the previously irradiated breast was associated with a significantly increased risk of complications, a finding that was consistent with our results.

However, differentiating between the risk of major and minor complications is an important factor in deciding the risk–benefit ratio of breast reduction surgery for women with a previously irradiated breast. Lorentzen et al used a different approach to classify major and minor complications. The authors defined major complications as those requiring or presumed to require treatment. Hence, all cases of fat necrosis, regardless of outcomes or severity, were considered “major complications.” Conversely, cases of wound dehiscence requiring reoperation were considered “minor wound healing problems.” Although a matter of semantics, this distinction plays a critical role in determining clinical decision. Our distinction between major and minor complications is one of the strengths of this study and is supported in the literature [41].

Furthermore, the review by Lorentzen et al. does not consider several recent, large-volume case series which have been published. Overall, their paper considered 107 patients, as compared to 188 in this review. This disparity in sample size could be attributed to their exclusion of small case series with fewer than five patients. This approach is supported by the increased risk of bias in small case series and case reports [42]. However, this is not a universal approach, and in an attempt to maximise statistical power, ensure comprehensive review and use quality assessment tools, the smaller studies were included in this review. However, their review is strengthened in its thorough methodology and discussion.

### Limitations

Although this study is strengthened in its increased statistical power, several limitations remain. We included data from 1990 to 2021, only fifteen studies were found to be eligible in our systematic review and most of these studies were case series and retrospective studies. The selected patient cohort limits a definitive conclusion due to biases in patient selection. Data are scarce regarding post-operative complications after reduction mammoplasty and mastopexy in previously irradiated breasts.

The lack of data on the severity of certain complications and their management means complications could not be clearly categorised. The last column of Table 6 shows *n*=21 complications whose severity is unknown. Hence, of 87 overall complications recorded, *n*=21 (24.1%) remained undifferentiated. Similarly, lack of clear data on asymmetry and aesthetic outcomes makes drawing conclusions from this systematic review challenging in this domain. The scarcity of data in this area indicates that most surgeons consider breast reduction surgery in irradiated breasts contradictory owing to the inherent risk of complications after reduction surgery in irradiated breasts [37].

The importance of patient-reported outcomes measures (PROMs) has been increasingly recognised as an important outcome measure. None of the case series reported PROMs and therefore was unable to be assessed in the current systematic review. Henceforth, it is suggested that oncologists/surgeons must counsel their patients about the increased risk of post-surgical complications and delayed healing of an irradiated breast as compared to a non-radiated counterpart. Furthermore, it is also suggested to have a careful and close follow-up of these patients in the post-surgical period [43, 44]. This is an area that requires further consideration in the medical literature. Finally, our review has not considered other pertinent factors in management such as chemotherapy, length of stay and patient satisfaction scores (Table 7).

**Table 7:** Quality assessment of included studies using the MINORS tool.

Author	A clearly stated aim	Inclusion of consecutive patients	Prospective collection of data	Endpoints appropriate to the aim of the study	Unbiased assessment of the study endpoint	Follow-up period appropriate to aim of the study	Loss to follow-up less than 5%	Prospective calculation of the study size	Total score
Handel et al, [15]	2	0	0	2	0	2	2	0	8
Spear et al, [32]	2	2	1	2	0	0	0	0	7
Kronowitz et al, [23]	2	2	1	2	0	2	0	0	9
Tuncer et al, [22]	2	0	0	2	0	2	2	0	8
Christiansen et al, [4]	0	2	0	1	0	0	0	0	3
Chin et al, [33]	2	2	0	2	0	2	2	0	10
Parret et al, [19]	2	2	0	2	0	2	2	0	10
Patel et al, [34]	2	2	1	2	1	2	1	0	11
Munhoz et al, [29]	2	2	0	2	0	2	2	0	10
Dal Cin et al, [43]	2	2	0	2	0	2	2	0	10
Spear et al, [45]	2	2	0	2	1	2	2	0	11
Egro et al, [30]	2	0	0	2	1	2	0	0	7
Weichman et al, [36]	2	0	0	2	0	2	0	0	6
Barnea et al, [44]	2	2	0	2	0	2	0	0	8
Prasidha et al, [40]	2	2	0	2	0	2	2	0	10

## Conclusion

The findings of our review imply that prior irradiation is significantly associated with an increased rate of minor complications after reduction mammoplasty and mastopexy. Despite non-significant statistical finding of the relative risk of experiencing a major complication in the IB, it is at least 2.5 times higher compared to NIB. The *p*-value of 0.06 while not significant is approaching statistical significance. We would therefore suggest that with future studies and more patients and therefore statistical power, major complications may possibly show statistical significance. With this in mind, we would advocate that surgeons take time to include this finding in their risk versus benefit discussions with their patients.

## Supplementary Information

Below is the link to the electronic supplementary material.

Supplementary file1 (PNG 11 KB)

Supplementary file2 (DOCX 12 KB)

Supplementary file3 (DOCX 12 KB)

## Data Availability

All extracted raw data may be accessed through contact of the corresponding author upon reasonable request.
