# Supplementary figures and images for: Does Prior Breast Irradiation Increase Complications of Subsequent Reduction Surgery in Breast Cancer Patients? A systematic Review and Meta-Analysis
Source: Aesthetic Plast Surg. 2024 Apr 24;48(21):4365–80. doi: 10.1007/s00266-024-04038-6 (PMC11588870; doi:10.1007/s00266-024-04038-6)

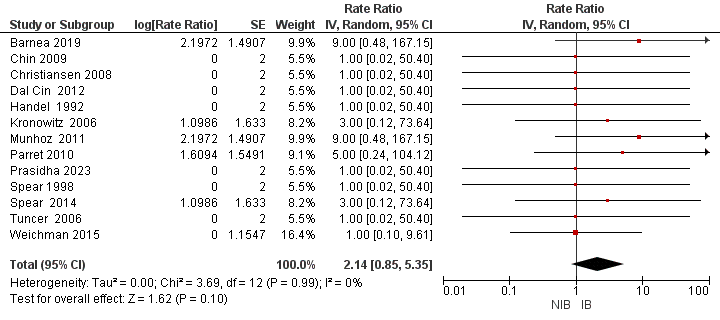

Supplement: Supplementary file 1 — Supplementary file1 (PNG 11 KB) [file 266_2024_4038_MOESM1_ESM.png]
