# Supplementary material for: Does Prior Breast Irradiation Increase Complications of Subsequent Reduction Surgery in Breast Cancer Patients? A systematic Review and Meta-Analysis
Source: Aesthetic Plast Surg. 2024 Apr 24;48(21):4365–80. doi: 10.1007/s00266-024-04038-6 (PMC11588870; doi:10.1007/s00266-024-04038-6)
Supplement: Supplementary file 2 — Supplementary file2 (DOCX 12 KB) [file 266_2024_4038_MOESM2_ESM.docx]

**Legend Supplementary figure**

**Supplementary Fig 1.** Forest plot depicting incidence of fat necrosis in the irradiated and nonirradiated breast.
