# Supplementary material for: Does Prior Breast Irradiation Increase Complications of Subsequent Reduction Surgery in Breast Cancer Patients? A systematic Review and Meta-Analysis
Source: Aesthetic Plast Surg. 2024 Apr 24;48(21):4365–80. doi: 10.1007/s00266-024-04038-6 (PMC11588870; doi:10.1007/s00266-024-04038-6)
Supplement: Supplementary file 3 — Supplementary file3 (DOCX 12 KB) [file 266_2024_4038_MOESM3_ESM.docx]

**Search Strategy**

MEDLINE/EMBASE/PubMED Search Terms

| 1. exp Breast Neoplasms/ or breast irradiation/ or mastopexy |
| --- |
| 2. (breast adj4 cancer*).mp. [mp=title, abstract, original title, name of substance word, subject heading word, keyword heading word, protocol supplementary concept word, supplementary concept word, unique identifier, synonyms] |
| 3. ((breast or mammary) (“breast cancer” or neoplasm* or carcinoma” or breast*) |
| 4. “breast conserving therapy” |
| 5. breast irradiation.mp. |
| 6. “breast reduction”.tiab. |
| 7. “mastopexy”.mp |
| 8. exp breast conserving therapy/ |
| 9. exp Breast or mastopexy |
| 10. 1 or 2 or 3 or 4 or 5 or 6 or 7 or 8 or 9 |
| 11. exp reduction mammaplasty |
| 12. “breast reduction”.mp. |
| 13. 1 or 2 or 3 or 4 or 5 or 11 or 12 |
